# Botulinum Toxin Type A for Glabellar Frown Lines: What Impact of Higher Doses on Outcomes?

**DOI:** 10.3390/toxins13070494

**Published:** 2021-07-16

**Authors:** Joely Kaufman-Janette, Sue Ellen Cox, Steven Dayan, John Joseph

**Affiliations:** 1 Skin Associates of South Florida, Skin Research Institute, 4425, Ponce De Leon Boulevard, Suite 200, Coral Gables, FL 33146-1871, USA; 2 Aesthetic Solutions, Chapel Hill, NC 27517, USA; sec@aesthetic-solutions.com; 3 DeNova Research, Chicago, IL 60611, USA; SDayan@DrDayan.com; 4 Clinical Testing of Beverly Hills, Encino, CA 91436, USA; drjohnjoseph@sbcglobal.net

**Keywords:** botulinum toxin, glabellar lines, glabellar rhytides, high dose, onabotulinumtoxinA, abobotulinumtoxinA, incobotulinumtoxinA, efficacy, safety, duration

## Abstract

Botulinum toxin serotype-A (BoNT-A) preparations are widely used to improve the appearance of wrinkles. While effective and well tolerated, patients require retreatment over time to re-establish the effects. There is growing interest from patients as to whether higher doses can prolong response without significantly increasing side effects. We reviewed the efficacy and safety evidence for high-dose BoNT-A treatment of glabellar lines, by evaluating high-dose studies published since 2015. Toxins approved for glabellar line treatment in the US or Europe were considered. “High-dose” indicated doses above the licensed dose for each BoNT-A preparation. Five studies met the inclusion criteria and most were randomized, double-blind trials; designs and population sizes varied. Findings suggested that higher-dose BoNT-A treatment is feasible and may improve response duration without increased safety issues. Around 9 months’ median duration was achieved with a 2–2.5-fold increase of the abobotulinumtoxinA on-label dose, or with a 5-fold increase in incobotulinumtoxinA dose. A 2–4-fold increase of the onabotulinumtoxinA on-label dose yielded a median duration of around 6 months. Importantly, patient satisfaction and natural look remained with increasing abobotulinumtoxinA doses. While more data are needed, these findings may lead to more effective, individually tailored treatment plans to meet patient expectations.

## 1. Introduction

Glabellar frown lines develop due to contraction of the corrugator, depressor supercilii and/or procerus muscles of the face, resulting in ‘brow furrows’, particularly between the eyebrows. Over time with repeated glabellar muscle complex contraction, these lines can become pronounced and even noticeable at facial repose. Their presence can often be perceived as a sign of aging and may erroneously give the impression of emotions such as anger or sadness [1,2,3]. For some people, the aesthetics of glabellar lines can negatively impact their psychological well-being, self-confidence and quality of life [3,4].

Botulinum toxin type A (BoNT-A) is a widely accepted treatment to improve the appearance of glabellar lines. BoNT-A is one of seven serotypes (A–G) of botulinum toxin, all of which are produced by the gram-positive anaerobic bacterium Clostridium botulinum [5]. Only serotype A is approved for cosmetic use including the treatment of glabellar lines [5]. BoNT-A acts by inhibiting release of the neurotransmitter acetylcholine from the presynaptic nerve terminal, thereby preventing muscular contraction [5,6]. When injected into the muscles of the glabellar complex, the result is a smoothing of the frown lines.

The cosmetic effect of BoNT-A preparations generally starts within a few days of injection and reaches maximum effect within 2–4 weeks. Thereafter, the effects decline gradually and, ultimately, the frown lines return to their original appearance, with patients requiring retreatment to re-establish effects. Generally, duration of effect after on-label treatment is 3–5 months [7,8,9,10,11,12,13,14] (Table 1). The need for frequent retreatment can be inconvenient for many individuals; this may be mitigated if it is possible to prolong the effect between standard injection rates. Thus, there is growing interest in the feasibility of using higher than on-label doses of BoNT-A to prolong its effects on the glabellar lines, although this needs to be balanced with potential changes to the safety profile.

Preclinical studies in mouse models have shown that time until recovery of leg muscle paralysis after injection of BoNT-A into the hind limb, assessed by voluntary running activity, is proportional to the amount of BoNT-A injected [15], supporting the rationale that increased dose of BoNT-A could lead to increased duration of action. The molecular rationale for a prolongation of effect is thought to be related to the amount of active neurotoxin core 150 kDa protein reaching receptors in the neuromuscular junction; greater amounts of active neurotoxin may result in prolonged block of neurotransmitter release [16]. All BoNT-A preparations contain the same active 150 kDa molecule albeit in differing amounts. There are greater amounts of core neurotoxin in the total dose of abobotulinumtoxinA (ABO; 0.27 ng) approved for treatment of glabellar lines than the total approved doses of onabotulinumtoxinA (ONA; 0.18 ng) and incobotulinumtoxinA (INCO; 0.08 ng)—which may influence their individual onset and duration of action characteristics [16].

Here, we review the most recent data on effectiveness and safety of high-dose BoNT-A for the correction of glabellar lines, limited to products already approved for the treatment of glabellar lines in the United States (US) or Europe, i.e., ABO, ONA, INCO and prabotulinumtoxinA (Table 1), based on articles published since 2015 and recently presented conference data.

## 2. Results

Five studies met the inclusion criteria. Table 2 summarizes study designs and common endpoints of responder rate, duration of response and safety for these studies. To date, no studies of prabotulinumtoxinA in glabellar lines investigating higher than the standard 20-unit (U) dose were identified; therefore, this BoNT-A preparation is not discussed.

### 2.1. Efficacy and Safety

#### 2.1.1. AbobotulinumtoxinA

ABO (Dysport^®^ [US]/Azzalure^®^ [Europe], manufactured as a powder-for-reconstitution formulation by Ipsen Biopharm Ltd. and co-marketed by Ipsen Biopharm Ltd. and Galderma Ltd.) received US and European approval in 2009 for the temporary improvement in the appearance of moderate to severe glabellar lines associated with procerus and corrugator muscle activity in adults <65 years of age [9,10]. The standard total dose for the treatment of glabellar lines is 50 U, divided into injections of 10 U each in five sites (Table 1).

Data from two studies of high-dose (up to 125 U) ABO for glabellar lines are reported. A prospective, open-label, duration-of-response pilot study of ABO 120 U (2.4-fold higher than on-label), in 30 subjects (23% male; 53% with severe [Grade 3] lines at baseline) reported high responder rates—proportion achieving a score of 0 or 1 on a 4-point categorical scale for line severity at maximum frown—ranging from 90% at Day 30 to 48% at Day 150 (21.4 weeks) [17] (Table 2). The median duration of response for all subjects, defined as time until return to baseline severity and estimated by Kaplan-Meier analysis, was 150 days (95% confidence interval [CI]: 120, 180 days) or 21.4 weeks. This was longer than had been reported for the on-label 50 U dose in pivotal Phase 3 clinical trials (median duration: 85 days [12.1 weeks] [22], 117 days [16.7 weeks] [23]). The authors found that ABO 120 U was particularly effective at reducing the appearance of moderate (Grade 2) glabellar lines with a median duration of response of 165 days (23.6 weeks) (95% CI: 90, 180 days). Subjects with severe (Grade 3) glabellar lines at baseline had a duration of response with ABO 120 U of median 75 days (10.7 weeks) (95% CI: 30, 120 days), and the authors suggested that this population may benefit from a further increased dose. Notably, no new safety signals were identified with the 120 U dose. Three reported treatment-related adverse events (AEs) (one each of dizziness, right corrugator twitch and brief glabellar muscle tightening) were mild in severity and transient, and there were no cases of eyelid or eyebrow ptosis in the study. The publication did not report the assessment of neutralizing antibodies.

A subsequent Phase 2, randomized, double-blind, placebo-controlled trial prospectively explored doses of ABO ranging from 50 to 125 U (2.5-fold higher than on-label), with preliminary results presented at Maui Derm 2021 [18]. A total of 399 subjects were enrolled (n~80 subjects per group; 12% male), of whom 68% had severe lines at maximum frown at baseline. Responder rates (proportion achieving ≥1-grade improvement from baseline at maximum frown) at Week 4 ranged from 98% to 100% across the ABO dose groups (Table 2). The study compared each ABO dose to placebo, but was not powered to determine statistical differences between different ABO doses. However, dose escalation tended to lead to higher response rates (ranging from 53% with the 50 U dose to 69% with the 125 U dose at Week 24). In addition, duration of response (defined as time to return to baseline from a none-or-mild response on both investigator- and subject-assessed scales) ranged from a median of 32.3 weeks with 50 U to 36.6 weeks with 125 U (Table 2). Severity improvement of ≥1-grade was maintained from baseline to Week 36 in around one-third (35% and 31%) of subjects who received 100 and 125 U doses, but was also maintained in 18% of subjects who received 50 U and 26% who received 75 U. In addition, none-or-mild responder rates of 5 to 12% in the 50 to 125 U dose groups at Week 36 supported the ≥1-grade improvement results. The incidence of AEs was low across all ABO doses and consistent with previous studies of the standard 50 U dose [1,22,23,24,25,26,27,28,29]. Eyelid ptosis was not dose-related and was reported by a total of 4 subjects treated with ABO (75 U [1 case, 1.3%], 100 U [2 cases, 2.5%], 125 U [1 case, 1.2%]) (Table 2); all cases were mild (n = 2) or moderate (n = 2) and transient, resolving without sequelae [18]. No subjects developed neutralizing antibodies after ABO treatment in any of the dose groups [18].

#### 2.1.2. OnabotulinumtoxinA

ONA (Botox Cosmetic^®^/Vistabel^®^, Allergan Inc) received approval in the US in 2002 and shortly thereafter in Europe for the temporary improvement in the appearance of moderate to severe glabellar lines associated with corrugator and/or procerus muscle activity in adults [11,12,14]. The standard total dose for the treatment of glabellar lines is 20 U divided into five equal intramuscular injections of 4 U each (Table 1).

One study of high-dose ONA treatment of glabellar lines met the inclusion criteria, with results recently presented at the virtual ASDS 2020 Annual Meeting [19] (Table 2). In this randomized, placebo-controlled, dose-ranging study in 226 women, the highest doses tested (40–80 U, up to 4-fold the on-label dose) of ONA demonstrated longer duration of response. The median time until return to baseline severity score at maximum frown among responders was approximately 24.0 weeks for 40–80 U, compared with a median of 19.7 weeks for the on-label 20 U dose (*p* ≤ 0.03 for each comparison of 20 U with a higher dose) [19] (Table 2). Notably, there was a plateau in terms of duration of response among the 40, 60 and 80 U groups (range: 24.0 to 24.1 weeks). Responder rate (proportion achieving ≥1 grade improvement from baseline at maximum frown) at Week 24 was increased with doses higher than 20 U, although there was no clear dose-dependent effect beyond the 40 U dose (39%, 31%, 32% and 16% for the 80, 60, 40 and 20 U groups, respectively; Table 2). At Week 28, the ≥1-grade improvement responder rates were around 10% (20 U) to 20% (40 to 80 U) and at Week 36 <10% for all dose groups. There was no dose-proportional effect on the ONA safety profile up to doses of 80 U, and no severe AEs. One of 7 subjects (14.3%) experienced mild eyelid ptosis (80 U group) during an open-label phase of the study (none during the double-blind phase) and one subject (2.0%) had mild eyebrow ptosis (20 U group) during the double-blind phase. Both resolved without sequelae. Madarosis was also reported in one subject (1.9%, 80 U) during the double-blind phase. The publication did not report the assessment of neutralizing antibodies.

#### 2.1.3. IncobotulinumtoxinA

INCO (Xeomin^®^ [US]/Bocouture^®^ [Europe], Merz Pharmaceuticals) was approved in 2010 in Europe (based on the UK SmPC) and in the US for the temporary improvement in the appearance of moderate to severe glabellar lines with corrugator and/or procerus muscle activity in adults [7,8]. The standard licensed dose of INCO for glabellar lines is 20 U per treatment divided into five equal intramuscular injections of 4 U each (Table 1).

Two INCO studies were found in the literature review; neither was a placebo-controlled trial. A Phase 2, randomized, double-blind trial investigated INCO 20, 50 and 75 U (up to 3.75-fold the on-label dose) in 151 subjects, of whom 87% were female and 85% had severe glabellar lines at maximum frown at baseline, on a 4-point facial wrinkle scale (FWS) [20]. Subjects were required to remain in the study for at least 180 ± 7 days (25.7 ± 1 weeks) (Table 2). The median duration of treatment effect (time until return to baseline severity among responders) was higher with the 75 and 50 U doses, compared with the standard dose (20 U) (30.0, 26.4 and 25.3 weeks, respectively). Even though not statistically powered to compare dose groups, Cox proportional hazard regression analysis suggested a dose effect on duration of response: hazard ratio (HR) = 0.67 for 75 vs. 50 U (95% CI: 0.46, 0.98; *p* = 0.0400), and HR = 0.56 for 75 vs. 20 U (95% CI: 0.34, 0.90; *p* = 0.0166). Nearly all responders (subjects achieving ≥1-grade improvement in FWS at maximum frown) in the 75 U group had not returned to baseline severity within the first 120 days (17.1 weeks). The incidence of treatment-related AEs was low in all dose groups (up to 13.1%) and there were no serious AEs. Treatment-related eyelid ptosis was reported in 2 subjects (1.3%); both events were transient. The publication did not report the assessment of neutralizing antibodies.

A second study—an investigator-initiated, Phase 4, randomized trial in only 50 subjects with moderate to very severe (based on the Merz Aesthetics 5-point scale) glabellar lines—also showed significantly longer median duration of effect (time until return to baseline severity at maximum frown) with higher doses of INCO among the 38 subjects who completed the study: 100 U, 38.6 weeks (95% CI: 34.3, 47.1; n = 17); 60 U, 25.7 weeks (95% CI: 25.7, 30.0; n = 13); 20 U, 17.1 weeks (95% CI: 12.9, 25.7; n = 8) [21]. In addition, Cox regression analysis indicated a significant effect of dose on time to relapse to baseline (*p* < 0.001, Wald chi-square test), with HRs indicating a longer time to relapse in the 60 vs. 20 U groups (HR = 0.21; 95% CI: 0.10, 0.43) and the 100 vs. 20 U groups (HR = 0.06; 95% CI: 0.10, 0.43). All AEs were mild in severity and there were no reports of ptosis. The publication did not report the assessment of neutralizing antibodies.

### 2.2. Time to Onset of Effect

The relationship between higher doses of BoNT-A and onset of BoNT-A effect on glabellar lines has been less studied than duration of response. Where it has been investigated, methodology has differed between trials with some using physician assessment and others using subject diaries. In general, time to onset of effect with standard licensed doses of BoNT-A preparations has been reported to occur within an average of 1–4 days after injection [23,29,30,31]. In the high-dose studies in this review, Joseph et al. reported an early onset of effect on glabellar lines for ABO, with a subject-reported median time to onset of 2 days after treatment, for all ABO doses from 50 to 125 U [18]. Polacco et al. reported a possible relationship between increasing dose of INCO (20, 60 and 100 U) and faster onset of action with a marked difference in the onset of action between the 60 and 100 U dose groups, although this was based on visual inspection of the data and was not formally assessed [21]. Further prospective studies are needed to clarify a dose relationship for time to onset for the different BoNT-A preparations.

### 2.3. Subject Satisfaction

Subject satisfaction was reported in two of the studies discussed in this review. For ABO, Joseph et al. reported high subject satisfaction (≥99% of subjects being satisfied or very satisfied with the aesthetic outcome) after treatment with all doses (50 to 125 U) at Week 4 based on questionnaire results, which was sustained in ≥82% up to Week 24 and ≥67% up to Week 36 for all ABO doses. Furthermore, ≥89% of subjects reported natural-looking results at all timepoints up to Week 36 across all ABO doses [18].

For ONA, Cox et al. reported that subject satisfaction with the effect of treatment on the facial lines at Week 24 increased from 20 U (24% satisfied subjects) to 40 U (60%) but thereafter decreased with further escalated doses (60 U, 51% and 80 U, 39%). Similar results were obtained for satisfaction with natural look [19].

## 3. Discussion

### 3.1. Do Higher Doses of BoNT-A Translate into a Longer Duration of Effect?

Standard licensed doses of BoNT-A provide a reasonably long duration of action in glabellar lines: up to 4 months for ONA and INCO, and up to 5 months for ABO, according to each Summary of Product Characteristics (SmPC) [7,8,9,10,11,12,13,14]. Note that the products differ in formulation and their units are not interchangeable. In a recent real-world observational study of 150 subjects receiving glabellar line treatment with the standard 50 U dose of ABO, the median time between treatments over three cycles was 5 months [25]. In addition, a duration of effect of up to 6 months after injection has been shown for the on-label dose of ABO in recent studies [28,32]. Nevertheless, there are potential benefits for subjects in further prolonging BoNT-A effects between injections, in terms of extended effect on glabellar frown lines and greater convenience due to less frequent injections and trips to the clinic for retreatment.

Overall, data from high-dose studies indicate that higher doses of BoNT-A are associated with enhanced efficacy and prolonged duration of effect over the on-label dose for each preparation. For ABO, Joseph et al. demonstrated that a single 120 U treatment (2.4 times the on-label dose) was associated with a high responder rate at Day 150 (Week 21.4) (48% none-or-mild severity) and a median duration of 5 months [17], as compared to 17% responder rate and a duration of up to 5 months at 50 U as reported in the SmPC. In addition, a subsequent Phase 2, randomized, controlled trial directly showed that 0.5–2.5-fold increases in ABO standard dose (i.e., 75–125 U) achieved a higher ≥1-grade responder rate at Week 24 than the on-label 50 U dose (58–69% vs. 53%) [18]. A 2–2.5-fold increase above the 50 U ABO on-label dose (to 100 or 125 U) extended the median duration of effect to approximately 9 months [18]. For ONA, increasing the dose by 2–4 times was associated with greater responder rates and longer duration of response than the standard 20 U dose [19]. A 4-fold increase in the ONA on-label dose (from 20 to 80 U) increased the median duration to around 6 months [19], although the same duration of response was observed at 40 and 80 U. Dose-ranging studies of INCO also suggest a trend toward increased duration of response with escalating doses; increasing the on-label dose by 3.75-fold (from 20 to 75 U) increased the median duration of response to 7 months [20] and a 5-fold increase of the on-label dose (to 100 U) increased the median duration to 9 months [21]. Another BoNT-A product, daxibotulinumtoxinA, under development for the treatment of glabellar lines, has been shown in Phase 3 studies using a dose of 40 U (planned registration dose) to achieve a median duration of response (time to return to baseline from a none or mild response) of 26.0–27.7 weeks (6.1–6.5 months) [33], which is in the same range as shown for 2.5-fold the on-label dose of INCO [21] and the on-label dose of ABO reported in Joseph et al. [18] (Table 2).

Direct comparison of studies and efficacy outcomes between BoNT-A preparations is limited for many reasons. Potency units of different BoNT-A preparations are non-interchangeable due to differences in manufacturing processes, excipients, formulations and the assays used to determine potency [34]. Studies differ in design, population studied, timing of visits and length of follow-up, etc. Furthermore, it has been shown that there are notably greater amounts of active neurotoxin 150 kDa molecule in the standard on-label dose of ABO than in on-label doses of ONA or INCO [16]. Indeed, this may in part explain the lesser fold-increase in on-label dose needed to extend duration of effect with ABO than ONA or INCO [18,19,20]. A standardization of the dose units or establishment of a conversion ratio between toxin products would facilitate comparison, but no consensus has been reached [35]. Dose-response curves are not parallel between BoNT-A products and therefore a set ratio cannot be expected [36].

In summary, higher doses of BoNT-A do appear to translate into a longer length of effect, achieving median response durations between 6 and 9 months with the highest doses tested. However, a duration of around 9 months was achieved with only a 2–2.5-fold increase in ABO dose, while INCO required a 5-fold increase to achieve this duration and ONA achieved only a 6-month duration despite a 4-fold increase in dose. Furthermore, subject satisfaction and a natural look was maintained with a 2- and 2.5-fold increase in ABO dose, while the satisfaction decreased for ONA when the dose was increased by 3-fold or more.

### 3.2. Is There a Ceiling Dose Beyond Which Increasing the Dose No Longer Leads to a Difference in Efficacy?

Even though somewhat confounded by different study designs, current data point to a potential BoNT-A ceiling dose for glabellar lines. However, it is likely that the ceiling dose is influenced by factors such as sex, muscle mass and age, since these are known to impact clinical efficacy [11,22,37,38,39,40].

For ABO, the Phase 2 study reported by Joseph et al. showed a possible plateau of effect with doses in the range of 2–2.5-fold higher than the on-label dose (median time to return to baseline glabellar line severity of 36.0 and 36.6 weeks with a 2-fold and 2.5-fold higher dose, respectively), although the responder rates did not show this with certainty [18]. The dose-ranging study with ONA, performed in women only, showed that doubling the on-label ONA dose (from 20 to 40 U) was sufficient to achieve an increase in response duration, with no enhancement seen by further increasing the dose up to 4 times the on-label dose (80 U) [19], also suggesting a possible ceiling dose. In contrast, a prior study of ONA (before 2015) using the same dose range in men showed no plateau in efficacy with doses ≥2-fold the on-label dose, and greatest benefits with a 4-fold higher dose (80 U) [39]. For INCO, the data on duration of response in the two reviewed high-dose studies did not reveal a ceiling dose within the studied dose range (up to 5-fold the on-label dose) [20,21]. Altogether, this suggests that ceiling doses may exist, but are likely different for men and women.

Another important factor to consider is time to onset of effect. Unfortunately, this endpoint has been infrequently studied in dose-ranging and high-dose trials of BoNT-A. Joseph et al. reported a median time to onset of 2 days for all ABO dose groups ranging from the on-label dose of 50 U to 125 U (2.5-times the on-label dose) [18], showing a rapid onset for all doses including the on-label dose. Most probably there is a physiological limit to how fast the effect of treatment is noticeable for the patient. In prior studies, a sex difference has been seen for onset time for ONA, ABO and INCO, with slightly faster onset seen in women [41,42], but this was not examined in the more recent high-dose studies.

Patient satisfaction should also be considered when evaluating the possibility of a plateau of response with increasing BoNT-A doses. In the ABO study evaluating doses from 50–125 U (up to 2.5-fold the on-label dose), high patient satisfaction was maintained throughout the highest dose level [18]. This can be compared to the ONA dose-ranging study using 20–80 U (up to 4-fold the on-label dose), where a decrease in patient satisfaction was observed when doses were escalated more than 2-fold higher than the on-label dose [19]. The effects on patient satisfaction when increasing the dose will be an important consideration when evaluating potential ceiling doses for each BoNT-A product and when planning the optimal treatment for an individual patient, since the ultimate goal of glabellar line treatment is to satisfy the patient’s expectations.

In summary, it seems likely that there is a ceiling dose of BoNT-A beyond which further increases in efficacy are not seen; however, further studies may be needed to define this dose for different BoNT-A preparations and in different patient populations.

### 3.3. Are Higher Doses of Botulinum Toxin in the Glabellar Lines Safe and Appropriate to Use on All Patients?

In the recent high-dose studies reviewed, all BoNT-A preparations were well tolerated over a range of doses, with no apparent dose-dependent increase in AEs compared with the standard dose, and no new safety concerns identified with doses higher than on-label, for all doses tested of ABO, ONA and INCO [17,18,19,20,21]. Of note, the doses in these studies were raised by decreasing the dilution volume of BoNT-A, thereby injecting a more concentrated solution. For ABO and INCO, injections in the studies were made using volumes of injection within the on-label range (0.05 or 0.08 mL for ABO; 0.05 mL for INCO), whereas ONA was injected using a smaller volume of injection (0.05 mL) in the studies compared to the on-label volume (0.1 mL) (Table 1 and Table 2). These findings are encouraging, as higher doses of BoNT-A could be expected to result in a higher incidence of AEs, the most common serious complication being blepharoptosis (drooping eyelid) as a result of toxin spread through the orbital septum to affect the lid elevator muscle [7,9,11,13]. To date, the incidence of ptosis has been low in high-dose BoNT-A studies for glabellar lines for all doses, including the on-label dose (Table 2). Furthermore, the incidence of ptosis did not appear to be dose-dependent, and most cases were mild and resolved without sequelae.

Development of neutralizing antibodies is a concern with BoNT-A injections and tends to increase with increasing BoNT-A dose and frequency of treatment [43]. However, typically doses used for aesthetic indications are much lower (e.g., 20–50 U) than doses used for adult therapeutic indications, which can reach 1000 U or more depending on the product and muscle(s) injected [7,8,9,10,11,12,13,14]. Previous analyses have shown that the rate of neutralizing antibody formation for BoNT-A used for therapeutic indications is low (2.1%) at all doses and even lower when used for aesthetic treatment of glabellar lines (0–0.4%) [44,45,46]. In the high-dose studies in this review, only one study reported measuring neutralizing antibodies (ABO [18]) and found no seroconversion after treatment with any of the doses. Further investigations are needed to confirm whether high doses of BoNT-A used for aesthetic treatment will lead to neutralizing antibody formation, particularly for ONA and INCO.

Higher doses of BoNT-A could potentially induce muscle atrophy, which has been reported to occur with BoNT-A treatment for aesthetic indications [47,48]. This has been suggested as a possible preferred effect for the glabellar indication [47] and was not studied in the high-dose studies included in this review.

These findings provide confidence in the use of higher doses of BoNT-A, as well as on-label doses, and support further studies investigating even higher doses in different populations.

### 3.4. Limitations

Limitations of this review include low numbers of patients in high-dose groups in some studies (INCO) and the fact that most studies were not powered to make statistical comparisons between dose groups. In addition, differences in study design—such as study population characteristics, absence or presence of a placebo group, follow-up period and definitions of responder rate and duration of response—mean no direct comparison between studies, or BoNT-A preparations, is possible.

## 4. Conclusions

This article reviewed high-dose BoNT-A treatment of glabellar lines and impact of dose increase on safety, duration of effect and satisfaction. Findings suggest that it is feasible to increase doses of BoNT-A above the standard dose without substantially increasing side effects. There were no new safety concerns identified with the highest doses of each BoNT-A preparation or dose-related increases in ptosis events. Higher doses were associated with longer duration of response, with a possible ceiling dose that needs further evaluation. A median duration of around 9 months was achieved with a 2—2.5-fold increase in ABO dose, and furthermore, high subject satisfaction and natural-looking results were obtained for all ABO doses. A 9-month median duration was achieved with a 5-fold increase in INCO dose, while a 2—4-fold increase in the ONA on-label dose only achieved a median duration of around 6 months. This could allow for BoNT-A injections to be given further apart than the current standard of retreatment every 3–5 months as needed. Further studies are warranted to investigate and support the use of higher doses or variable doses of BoNT-A for glabellar lines, with the aim of developing treatment plans tailored for the individual.

## 5. Materials and Methods

### Literature Search

A search of relevant studies was conducted in MEDLINE/PubMed. Search terms were limited to BoNT-A preparations that are approved in the US or Europe for the treatment of glabellar lines, and included “onabotulinum*”, “abobotulinum*”, “incobotulinum*”, “prabotulinum*, “botulinum toxin”, “botulinum toxin type”, “botox”, “vistabel”, “dysport”, “azzalure”, “bocouture”, “xeomin”, “jeuveau” and “glabellar lines”. Related terms were combined using the Boolean “OR” and “AND”. Studies were included if: (1) subjects were treated specifically for glabellar lines (studies were excluded if, in addition to glabellar lines, other facial wrinkles were treated at the same time); (2) they assessed doses of BoNT-A higher than the standard licensed dose; (3) reports were written in the English language. Studies were excluded if they were published before 2015. Recent congress presentations of high-dose BoNT-A use for glabellar lines that the author was aware of were also included. In addition, the bibliographies of included reports were reviewed to ensure no key studies were missed.

## Figures and Tables

**Table 1:** BoNT-A preparations currently approved in the US or Europe for treatment of moderate to severe glabellar lines in adults [7,8,9,10,11,12,13,14].

Generic (Trade Name[s])	Manufacturer/First Approved for Glabellar Lines	Dose (U)/no. Injection Points	Standard Total Dose for Glabellar Lines (U)	Volume (mL) Per Injection Point	Duration of Effect (as Stated in the License)
OnabotulinumtoxinA (Botox Cosmetic^®^/Vistabel^®^)	Allergan /2002	4/5	20	0.1	Approx. 3–4 months
AbobotulinumtoxinA (Dysport^®^/Azzalure^®^)	Ipsen/2009	10/5	50	0.05, 0.08 (US), 0.05, 0.1 (Europe)	Up to 5 months
IncobotulinumtoxinA (Xeomin^®^/Bocouture^®^)	Merz/2010 ^a^	4/5	20	0.04 to 0.1	Up to 4 months
PrabotulinumtoxinA (Jeuveau^®^)	Evolus/2019	4/5	20	0.1	Not stated

^a^ According to UK SmPC. Approx., approximately; U, standard potency unit (not interchangeable between products); US, United State.

**Table 2:** Studies investigating high doses of BoNT-A for the treatment of moderate-to-severe (GLS score 2–3) glabellar lines.

	ABO			ONA			INCO			
	[17]	[18]		[19]			[20]		[21]	
**STUDY DESIGN**										
Dose, U	120	50, 75, 100, 125		20, 40, 60, 80			20, 50, 75		20, 60, 100	
Increase vs. on-label dose	2.4-fold	Up to 2.5-fold		Up to 4-fold			Up to 3.75-fold		Up to 5-fold	
Volume of injection per injection point	0.08 mL	0.05 mL		0.05 mL			0.05 mL		0.05 mL	
Type of trial	Open-label, single-arm, pilot (IIS)	Phase 2, randomized, placebo-controlled		Randomized, placebo-controlled			Phase 2, randomized		Phase 4, randomized (IIS)	
No. of subjects	30	399 (~80 per group)		226			151		38 ^b^	
Females, %	77	88		100			87		82 ^b^	
Severe GLS ^a^ at baseline, %	53	68		60			85		–	
Follow-up period, months	~10 (300 days)	~9 (36 weeks)		~11 (48 weeks)			~6–12 ^c^		12	
**EFFICACY**										
Scale	4-point categorical scale	4-point photographic scale		4-point Facial Wrinkle Scale			4-point Facial Wrinkle Scale		5-point Merz Scale	
≥2-grade improvement + composite responder ^d^, %	Day 30 (Week 4) 120 U: 63	*Week 4* PBO: 3 50 U: 80 ^‡^ 75 U: 89 ^‡^ 100 U: 90 ^‡^ 125 U: 95 ^‡^		Not reported			Not reported		Not reported	
≥1-grade improvement from baseline, %	*Day 150 (Week 21.4)* 120 U: 62	*Week 24* PBO: 5 50 U: 53^‡^ 75 U: 65^‡^ 100U: 58^‡^ 125 U: 69^‡^	*Week 36* PBO: 0 50 U: 18 ^‡^ 75 U: 26 ^‡^ 100 U: 35 ^‡^ 125 U: 31 ^‡^	*Week 24* PBO: 0 20 U: 16 40 U: 32 * 60 U: 31 NS 80 U: 39 *	*Week 28* ^e^ PBO: 0 20 U: ~10 40 U: ~20 * 60 U: <20 NS 80 U: ~20 *	*Week 36 ^e^* PBO: 0 20 U: <10 40 U: <10 NS 60 U: <10 NS 80 U: <10 NS	No responder rates reported for INCO			
							*Week 24* *(Day 170, estimated from Kaplan-Meier graph)* 20 U: not shown 50 U: ~76 75 U: ~83	*Week 36* *(Day 250 estimated from Kaplan-Meier graph)* 20 U: not shown 50 U: ~5 75 U: ~15	*Week 24* *(Day 170, estimated from Kaplan-Meier graph)* 20 U: ~14 ^f^ 60 U: ~81 ^f^ 100 U: ~100 ^f^	*Week 36* *(Day 250 estimated from Kaplan-Meier graph)* 20 U: 0 ^f^ 60 U: ~19 ^f^ 100 U: ~55 ^f^
Median time to return to baseline severity, weeks	120 U: 21.4 ^g^	50 U: 32.3 ^h^ 75 U: 34.3 ^h^ 100 U: 36.0 ^h^ 125 U: 36.6 ^h^		PBO: 9.1 ^i^ 20 U: 19.7 ^i^ 40 U: 24.1 ^i^ 60 U: 24.1 ^i^ 80 U: 24.0 ^i^			20 U: 25.3 ^j^ 50 U: 26.4 ^j^ 75 U: 30.0 ^j^		20 U: 17.1 ^f,k^ 60 U: 25.7 ^f,k^ 100 U: 38.6 ^f,k^	
**SAFETY**										
Safety summary	3 drug-related AEs, all mild in severity and transient	Generally similar across dose groups; no drug-related SAEs		Similar between dose groups			Generally similar across dose groups; no SAEs		All AEs were mild with no apparent dose-proportional effect	
Ptosis AEs	No lid or brow ptosis	4 cases of lid ptosis (2 mild, 2 moderate); all resolved; 1.3% of subjects (75 U), 2.5% (100 U), 1.2% (125 U)		*Open-label phase* 1 lid ptosis; 14.3% of 7 subjects (80U), mild, resolved *Double-blind phase* 1 brow ptosis; 2.0% (20U), mild, resolved			2 cases of lid ptosis; 1.3% of subjects overall (dose groups not reported)		No ptosis reported	

Standard doses are marked bold; ^‡^ *p* < 0.001 vs. placebo; * *p* < 0.05 vs. standard dose of 20 U; a.—Assessed by investigator/trained observer at maximum frown, b.—n = 50 patients were randomized and treated at least once; efficacy analysis was based on the 38 subjects who completed the study (subjects who dropped out after treatment were not analyzed), c—Subjects were required to remain in the study for a minimum of 180 ± 7 days and a maximum of 360 ± 7 days, d.—Severity grade of none or mild at maximum frown on both investigator- and subject-assessed scales concurrently, e.—Responder rates at Week 28 and Week 36 for ONA were estimated from the graph presented in the poster, f.— Severity improvement was evaluated on a 5-point scale, g.—Median time until return to score 2 or 3 among responders (defined as ≥2-grade improvement on a 4-point categorical scale from baseline), based on Kaplan-Meier analysis, h.—Median time until return to baseline from a none or mild (score 0 or 1) response, i.—Median time until return to baseline in Week 4 responders (defined as ≥1-grade improvement from baseline at Week 4), j.—Median time until return to baseline in responders (defined as ≥1-grade improvement from baseline), k.—Median time until return to baseline score from individual’s investigator-graded maximal contraction score. ABO, abobotulinumtoxinA; AE, adverse event; CI, confidence interval; GLS, glabellar line severity; IIS, investigator-initiated study; INCO, incobotulinumtoxinA; NS, not statistically significant; ONA, onabotulinumtoxinA; PBO, placebo; U, potency units (not interchangeable between products).

## Data Availability

No new data were created or analyzed in this study. Data sharing is not applicable to this article.
